# *Arabidopsis* guard cell CO_2_/HCO_3_^−^ response mutant screening by an aequorin-based calcium imaging system

**DOI:** 10.1186/s13007-020-00600-w

**Published:** 2020-04-29

**Authors:** Mengmeng Tang, Xiaowei Zhao, Yinling Hu, Miaomiao Zeng, Kai Wang, Nannan Dong, Xiaonan Ma, Ling Bai, Chun-Peng Song

**Affiliations:** 1 State Key Laboratory of Crop Stress Adaptation and Improvement, School of Life Sciences, Henan University, Kaifeng, 475004 China; 2 Biocover Technology Development Co., Ltd, Shixing Street, Shijingshan District, Beijing, 100195 China

**Keywords:** CO_2_/HCO_3_^−^, Stomatal movement, Aequorin (AEQ), Ca^2+^, High-throughput screening, *Arabidopsis thaliana*

## Abstract

**Background:**

The increase in atmospheric CO_2_ is causing a number of changes in plant growth such as increases in leaf area and number, branching, plant size and biomass, and growth rate. Despite the importance of stomatal responses to CO_2_, little is known about the genetic and molecular mechanisms that mediate stomatal development and movement in response to CO_2_ levels. Deciphering the mechanisms that sense changes in CO_2_ and/or HCO_3_^−^ concentration is critical for unraveling the role of CO_2_ in stomatal development movement. In *Arabidopsis*, CO_2_-induced stomatal closure is strongly Ca^2+^-dependent. To further dissect this signaling pathway and identify new components in the CO_2_ response pathway, we recorded [Ca^2+^]_cyt_ changes in mutagenized *Arabidopsis* leaves and screened for mutants with abnormal guard cell behavior in response to CO_2_/HCO_3_^−^.

**Results:**

We observed that 1 mM HCO_3_^−^ induces [Ca^2+^]_cys_ transient changes in guard cells and stomatal closure both in light and darkness. The changes in [Ca^2+^]_cys_ induced by HCO_3_^−^ could be detected by an aequorin-based calcium imaging system. Using this system, we identified a number of *Arabidopsis* mutants defective in both [Ca^2+^]_cyt_ changes and the stomatal response to CO_2_/HCO_3_^−^.

**Conclusions:**

We provide a sensitive method for isolating stomatal CO_2_/HCO_3_^−^ response genes that function early in stomatal closure and that have a role in regulating [Ca^2+^]_cyt_. This method will be helpful in elucidating the Ca^2+^-dependent regulation of guard cell behavior in response to CO_2_/HCO_3_^−^.

## Background

The stomata, which are formed by pairs of guard cells, can be considered the gas-exchange valves of plants. Stomatal aperture is regulated by several factors including phytohormone levels, carbon dioxide (CO_2_) concentration, humidity, light, and pathogens.

A higher ambient CO_2_ concentration increases leaf intercellular CO_2_ concentration and mediates stomatal closure in plants, whereas a lower CO_2_ concentration triggers stomatal opening. CO_2_ influences not only the stomatal response, but also the number of stomata per unit leaf. This number is decreasing due to the long-term effect of continuing CO_2_ concentration increases [1].

Despite the importance of stomatal responses to CO_2_, little is known about the genetic and molecular mechanisms mediating stomatal development and movement in response to elevation in CO_2_. CO_2_ levels have been increasing steadily, and it is estimated that atmospheric CO_2_ will reach 550 ppm in 2050 compared with 400 ppm presently [2], so it is increasingly urgent to discover the underlying mechanisms of guard cell regulation in response to CO_2_ levels.

CO_2_ sensing in animals is mainly linked to α-carbonic anhydrases (α-CAs) [3], which are also important for CO_2_ perception in fungi [3, 4]. Carbonic anhydrases (CAs) can accelerate the conversion of CO_2_ into HCO_3_^−^ and H^+^, which in turn induce related responses. In plants, CO_2_ also can be converted into HCO_3_^−^ and H^+^ by anhydrases [5]. The key question in understanding stomatal movement in response to CO_2_ is the mechanism for perception of changes in CO_2_ and/or HCO_3_^−^ concentration. Despite the importance of anhydrase enzymes in CO_2_ perception in mammalian and fungal systems [3, 4], no orthologous α-CAs has been identified in plants. There are six β-CAs in *Arabidopsis thaliana*. CA1- and CA4-related stomatal movements were controlled by CO_2_ in guard cells, whereas a *ca1 ca4* double mutant exhibited insensitive stomatal movement response to CO_2_ [6]. Expression of a mammalian α-CA in the *ca1 ca4* double mutant restored the stomatal response to CO_2_, implying that CA-mediated CO_2_ catalysis to HCO_3_^−^ and H^+^ in guard cells is the key step for transmission of the CO_2_ signal [6].

Through isolation and analysis of genetic mutants, a number of proteins have been identified that function in CO_2_-controlled stomatal movement, including the SLAC1 anion channel [7, 8], the PATROL1 Munc 13-like protein [9], the AtALMT12/QUAC1 R-type anion channel [10], and the RHC1 MATE transporter [11]. The characterization of these proteins has contributed to our understanding of the mechanisms of CO_2_-regulated guard cell behavior. For example, transporter protein RHC1 acts as a bicarbonate sensor, and the high-CO_2_-induced stomatal closure mediated by RHC1 is controlled by inhibition of HT1 (HIGH LEAF TEMPERATURE1) activity [11, 12].

HT1 is regarded as a negative regulator in the CO_2_ signaling pathway: it functions by promoting phosphorylation of OST1 and thus inhibiting its kinase activity [11]. Furthermore, OST1 protein kinase has been proved essential for high-CO_2_-induced stomatal closure [13, 14]. But still, many points remain controversial, such as the mechanism underlying CO_2_ sensing; the identities of the CAs involved in this pathway; the function of CAs under low-CO_2_ conditions; and the interaction of CO_2_ with light, temperature, humidity, and phytohormones in influencing stomatal movement.

The primary requirement for solving these questions is the isolation of mutants. Screening dependent on thermal imaging is quite common for isolating *Arabidopsis* mutants with abnormal guard cell behavior. Almost all of the mutants obtained until now, including *ht1*, *rhc1*, and *patrol1*, were obtained using this method. Although this method has been effective in unraveling the regulation network in CO_2_-mediated stomatal movement, it is still not clear of this regulation network; thus, it is urgent to develop new screening methods.

Calcium ion (Ca^2+^) has been shown to act as a key cellular second messenger in numerous plant processes. In *Arabidopsis thaliana*, abscisic acid (ABA), hydrogen peroxide, cold, and CO_2_ all can stimulate cytosolic Ca^2+^ ([Ca^2+^]_cyt_) oscillation, which causes stomatal closure [15]. CO_2_-induced stomatal closure is strongly Ca^2+^-dependent in *Arabidopsis*, consistent with previous findings in *Commelina* guard cells [16–18]. Cytosolic Ca^2+^ regulates stomatal closure by two mechanisms: short-term Ca^2+^-reactive closure and long-term Ca^2+^-programmed closure [15].

Extracellular CO_2_ induces changes of the [Ca^2+^]_cyt_ in *Arabidopsis* guard cells. To further dissect this signaling pathway, new components in the CO_2_ response pathway that are related to the [Ca^2+^]_cyt_ changes need to be identified. Here, we used a novel approach for screening genetic mutants to identify proteins involved in CO_2_ response. In this study, we used the Ca^2+^ reporter aequorin (AEQ) to record [Ca^2+^]_cyt_ changes in *Arabidopsis* leaves in real time in order to visualize locally induced [Ca^2+^]_cyt_ elevations in response to CO_2_ or HCO_3_^−^ stimulus. Although this screening method had already been used for analyzing the responses of *Arabidopsis* to different stimuli such as salt stress, ABA, sorbitol, and cold [15], it had not been tried for screening mutants with altered stomatal responses to CO_2_ or/and HCO_3_^−^. By using this system, we obtained several *mci* (mutant of HCO_3_^−^/CO_2_insensitive) and *mcs* (mutant of HCO_3_^−^/CO_2_sensitive) mutants. Further study with these mutants will be helpful for uncovering the mechanism for calcium-dependent CO_2_-regulated guard cell movement.

## Results

### [Ca^2+^]_cys_ changes induced by HCO_3_^−^ can be detected by an aequorin-based calcium imaging system

In our first experiment, we tested whether AEQ-transgenic Arabidopsis plants could be used to detect [Ca^2+^]_cys_ changes induced by HCO_3_^−^. As it is already known that the pH of incubation buffer (50 mM KCl, 0.1 mM CaCl_2_, 10 mM 2-(N-morpholino) ethanesulfonic acid (MES) and 10 μM coelenterazine) cannot be stabilized at 7.0 when the concentration of KHCO_3_ is above 5 mM, a lower concentration (1 mM) that has previously been used for analyzing guard cell behavior [2] was selected to avoid the putative influence of pH.

**Fig. 1:**
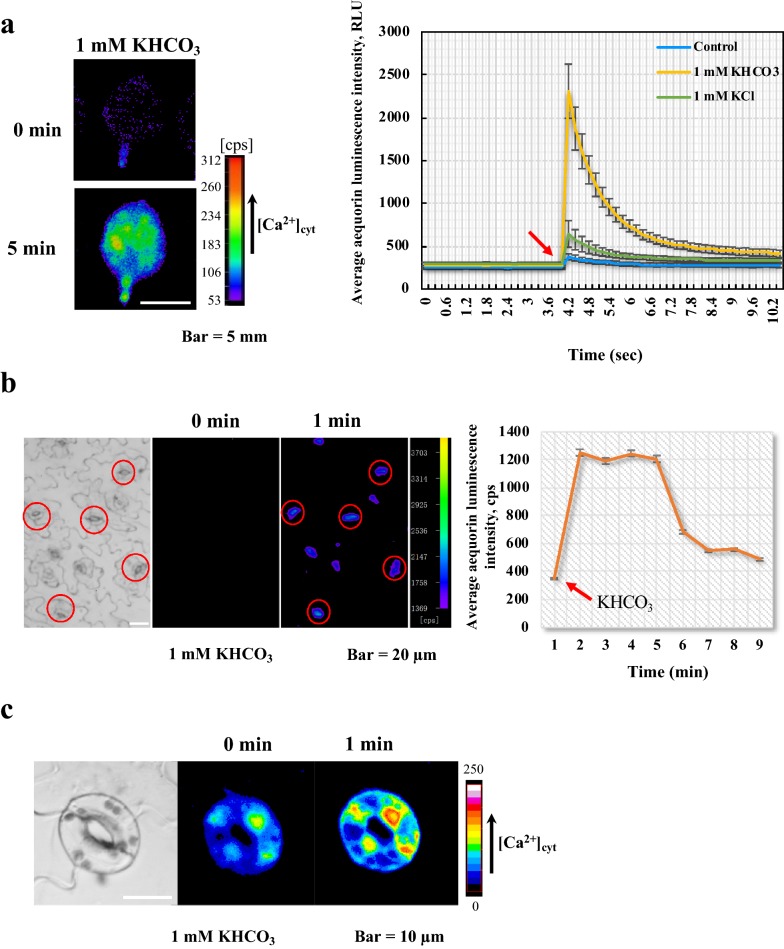
HCO_3_^−^-induced [Ca^2+^]_cys_ increase in *Arabidopsis* leaves and guard cells. **a** HCO_3_^−^-induced [Ca^2+^]_cys_ increase in *Arabidopsis* leaves. (Left) AEQ-transgenic *Arabidopsis* leaves were treated with 1 mM KHCO_3_, and analyzed by AEQ imaging at 0 and 5 min. (Right) Time-course analysis of [Ca^2+^]_cys_ changes after treatment with incubation buffer, 1 mM KCl and 1 mM KHCO_3_. Leaves were put individually into the wells of a 96-well plate and treated with incubation buffer, 1 mM KCl or 1 mM KHCO_3_. Luminescence recording began 4 s before treatment and was conducted at intervals of 0.2 s for a total of 12.4 s. Data for 59 leaves are shown (mean ± SE; n = 59). Bar = 5 mm. RLU, relative luminescence units. **b** HCO_3_^−^-induced [Ca^2+^]_cys_ increase in *Arabidopsis* guard cells. (Left) AEQ images of AEQ-transgenic *Arabidopsis* epidermal strips after 1 mM KHCO_3_ treatment. Red circles indicated guard cells. (Right) Time-course analysis of [Ca^2+^]_cys_ changes after 1 mM KHCO_3_ treatment. The luminescence data were quantified from guard cell pairs (red circles) in the left side of the figure (n = 8). Bar = 20 µm. **c** Emission images (FRET-dependent Venus, 526–536 nm; CFP, 473–505 nm) of epidermal strips expressing YC3.6 were taken before and 1 min after addition of 1 mM KHCO_3_ solution. Bar = 10 µm

The AEQ-transgenic *Arabidopsis* leaves were treated with 1 mM KHCO_3_, and after 5 min, dramatic increases in [Ca^2+^]_cys_ were detected in the leaves by analyzing the AEQ luminescence image (Fig. 1a, left). The average luminescence values increased from about 200 to 2300 RLU (Relative luminescence units, which represents the electrical signal values generated by stimulated photons) within 2 s of KHCO_3_ addition (Fig. 1a, right). We also added incubation buffer and same concentration of KCl as controls, and found that these only caused small changes of calcium (Fig. 1a, right). These suggested that 1 mM KHCO_3_ is effective for checking the cytosolic calcium changes with the AEQ system. Furthermore, when we analyzed individual guard cells within leaf epidermal strips under 1 mM KHCO_3_ treatment, a visible increase in luminescence of the guard cell was found after 1 min; when luminescence values were collected continuously for about 10 min, the quantified data of luminescence images of the guard cells confirmed the visually increase in [Ca^2+^]_cys_ to KHCO_3_ (Fig. 1b).

To further validate the AEQ-based screening method, we adopted another Ca^2+^ indicator, yellow Cameleon 3.6 (YC3.6), for measuring CO_2_/HCO_3_^−^-induced [Ca^2+^]_cyt_ increases in guard cells. The YC3.6 transgenic plants showed a marked increase in [Ca^2+^]_cyt_ when treated with 1 mM KHCO_3_. This was consistent with the results of AEQ, suggesting that the aequorin-based system is a reliable method of [Ca^2+^]_cyt_ measurement (Fig. 1c).

### 1 mM HCO_3_^−^ induces closure of *Arabidopsis thaliana* stomata whether in light or darkness

Since the aequorin-based system requires that samples first be incubated in incubation buffer in the dark for several hours, we measured the stomatal responses in both light and darkness. For this experiment, detached leaves of 3-week-old plants were incubated in a glass chamber with quicklime to remove CO_2_ from the chamber.

**Fig. 2:**
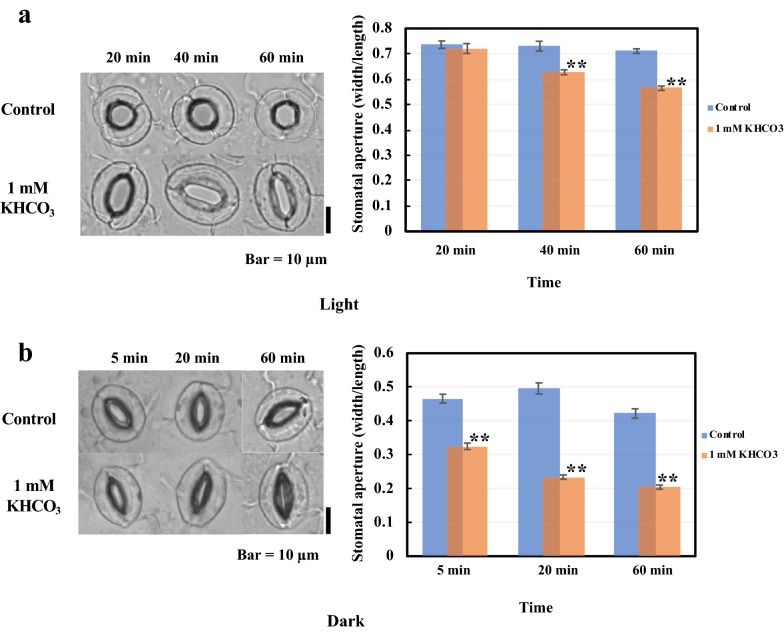
KHCO_3_ can induce stomatal closure in *Arabidopsis thaliana* whether in light or darkness. **a** KHCO_3_ induces stomatal closure under light. (Left) Guard cell images 20, 40, and 60 min after addition of 1 mM KHCO_3_. (Right) Changes in the apertures (width/length) of stomatal pores in response to 1 mM KHCO_3_. Data from three independent experiments are shown (mean ± SE; n ≈ 100 stomata; **P < 0.01, Student’s *t*-test). Bar = 10 µm. **b** KHCO_3_ induces stomatal closure under darkness. (Left) Guard cell images 5, 20, and 60 min after addition of 1 mM KHCO_3_. (Right) changes in the apertures (width/length) of stomatal pores in response to 1 mM KHCO_3_. Data from three independent experiments are shown (mean ± SE; n ≈ 100 stomata; **P < 0.01, Student’s *t*-test). Bar = 10 µm

After 2 h of incubation in the chamber under light conditions, almost all stomata had opened very well. The opened stomata were closing after 20 min of 1 mM KHCO_3_ treatment, and 60 min later, the stomatal apertures (width/length) were 0.71 ± 0.01 and 0.56 ± 0.01 for control and treated leaves, respectively (Fig. 2a).

Compared with the responses in light, the stomatal apertures were less after incubation in darkness without CO_2_. However, the stomata closed 5 min after 1 mM KHCO_3_ was added, and stomatal aperture decreased to 0.20 ± 0.01 for treated leaves at 60 min; while stomatal aperture was 0.42 ± 0.01 for the untreated at this time (Fig. 2b). The results showed that HCO_3_^−^-induced stomatal closure whether in light or darkness; thus, by using the aequorin-based system, it will be possible to indentify abnormal-response mutants for both stomatal movement and [Ca^2+^]_cys_ transient change at 1 mM KHCO_3_.

### High-throughput screening for CO_2_/HCO_3_^−^ response mutants

**Fig. 3:**
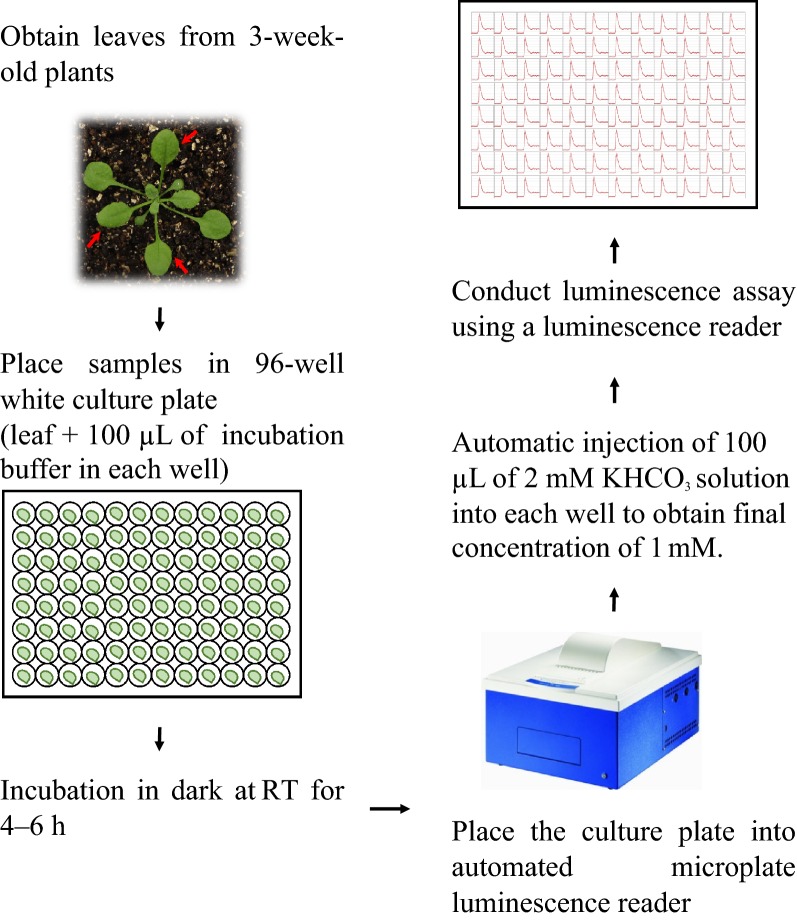
High-throughput strategy for isolation of CO_2_/HCO_3_^−^ response mutants. Schematic of the screening strategy with 96-well culture plates. The leaves (red arrows) of 3-week-old AEQ-transgenic *Arabidopsis* were placed in a 96-well culture plate and 100 µL of incubation buffer was added to each well. Plates were incubated in the dark at 25 °C for 4 to 6 h. The wells were automatically injected with 100 µL of 2 mM KHCO_3_ (to give a final concentration of 1 mM), and AEQ luminescence was recorded for each well

For high-throughput genetic screening with the aequorin-based system, we used the protocol shown schematically in Fig. 3. About 5000 AEQ-expressing *Arabidopsis* seeds were treated with 0.3% (w/v) ethyl methane sulfonate (EMS) and sown on soil. M_2_ seeds were collected individually and screened as described in Fig. 3. The leaves of 3-week-old M_2_ plants were placed in a 96-well plate and 100 µL of freshly prepared incubation buffer was added to each well for 4-6 h. AEQ luminescences of leave treatment with 1 mM KHCO_3_ were then identified by using a luminescence reader (LB960, Berthold) (Fig. 3). So far, approximately 35,000 M_2_ plants have been screened, and about 120 sensitive and 80 insensitive putative mutants have been identified.

The selected plants were examined further for their stomatal response to KHCO_3_ to narrow down the target mutants. HCO_3_^−^/CO_2_-induced stomatal closure of the putative mutants was assayed in M_2_ and again in M_3_, 6 out of 80 putative mutants with lower luminescence showed an insensitive stomatal response to HCO_3_^−^/CO_2_, and 4 out of 120 putative mutants with higher luminescence displayed a hypersensitive response.

### Characterization of mutants obtained by the aequorin-based screening method

By using the aequorin-based screening procedure, we identified HCO_3_^−^/CO_2_ response mutants that appeared abnormal in both [Ca^2+^]_cys_ and stomatal movement. To examin the stability of the mutants obtained by this method, two were selected for further analysis and named *mci1* (insensitive response) and *mcs1* (hypersensitive response).

**Fig. 4:**
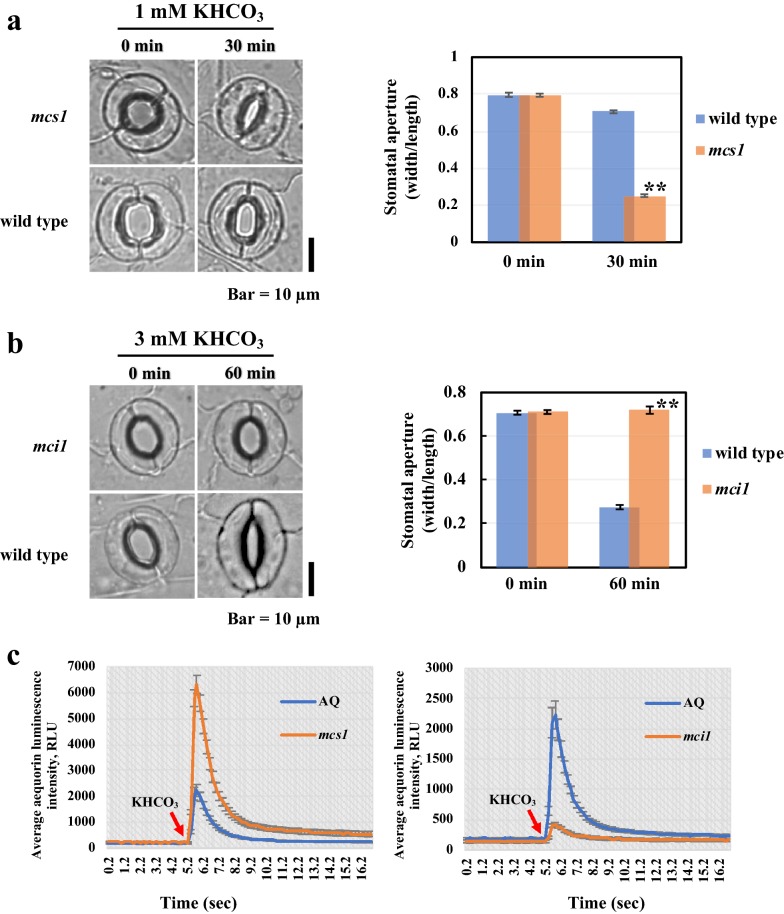
*mcs1* and *mci1* exhibited abnormal responses to HCO_3_^−^/CO_2_ with respect to [Ca^2+^]_cys_ changes and stomatal movement. **a** The *mcs1* mutant is hypersensitive to HCO_3_^−^/CO_2_ treatment. (Left) Images of wild-type and *mcs1* epidermal strips were taken, and guard cell images before and 30 min after addition of 1 mM KHCO_3_ are shown. (Right) Changes in the apertures (width:length) of stomatal pores in wild type and *mcs1* in response to 1 mM KHCO_3_. Data from three independent experiments are shown (mean ± SE; n ≈ 100 stomata; **P < 0.01, Student’s *t*-test). Bar = 10 µm. **b** The *mci1* mutant is insensitive to HCO_3_^−^/CO_2_ treatment. (Left) Images of wild-type and *mci1* epidermal strips were taken, and guard cell images before and 60 min after addition of 3 mM KHCO_3_ are shown. (Right) Changes to the apertures (width/length) of stomatal pores in wild type and *mci1* in response to 3 mM KHCO_3_. Data from three independent experiments are shown (mean ± SE; n ≈ 100 stomata; **P < 0.01, Student’s *t*-test). Bar = 10 µm. **c***mcs1* (left) and *mci1* (right) exhibited abnormal AEQ luminescence intensities changes in response to 1 mM KHCO_3_. Leaves were put individually into the wells of a 96-well plate, and luminescence values were recorded at intervals of 0.2 s after 1 mM KHCO_3_ was added. Data for 59 leaves are shown (mean ± SE). Orange lines indicate mutants; blue lines indicate wild type (AQ). RLU, relative luminescence units

We first monitored the stomatal movement of *mci1* and *mcs1* in response to HCO_3_^−^/CO_2_.The results clearly showed that 1 mM HCO_3_^−^ could induce stomatal closure within 30 min in *mcs1* but not in wild type (Fig. 4a). For *mci1*, even 3 mM HCO_3_^−^ could not induce stomatal closure after 1 h (Fig. 4b).

Consistent with the results of the screen, by comparing with wild type, AEQ luminescence intensities increased dramatically in *mcs1*, while no significant change was observed in *mci1* in response to HCO_3_^−^ treatment (Fig. 4c). These results further suggest that the products of *MCS1* and *MCI1* participate in HCO_3_^−^ signal transduction pathways regulating both [Ca^2+^]_cys_ and stomatal movement.

It is necessary to make sure that only a single gene locus functions in controlling a phenotype of interest before conducting gene mapping. After crossing each of the mutants with wild type, we analyzed the segregation of the F_2_ progeny. Phenotypes of F_2_ plants showed 3:1 (wild-type:*mci1* or *mcs1*) segregation, suggesting that *mci1* or *mcs1* was resulted from a recessive mutation. These two mutants are appropriate for subsequent gene mapping work.

Together, these data demonstrated that the high-throughput methods developed in this study are valuable for identifying new calcium-related components in the HCO_3_^−^/CO_2_-mediated stomatal closure signaling network pathway.

## Discussion

CO_2_ influences both stomatal movement and stomatal development; however, the mechanisms of guard cell perception and transduction are still not fully clear, and the sensors that mediate CO_2_-controlled stomatal movement remain enigmatic. Previous studies have suggested that intracellular bicarbonate acts as a second messenger in guard cells involved in mediating CO_2_ signal transduction [19–21]. To date, a number of proteins with critical roles in this signaling pathway have been identified, such as CA1 and CA4, HT1, SLAC1, RHC1, and others.

Because Ca^2+^ is a key cellular second messenger, transient change in [Ca^2+^]_cyt_ reflects most physiology processes including CO_2_-regulated guard cell behavior. GROWTH CONTROLLED BY ABSCISIC ACID 2 (GCA2) has been proved to function downstream of both CO_2_ signaling and ABA signaling by regulating [Ca^2+^]_cyt_. *gca2* mutant plants display decreased sensitivity of stomata to elevated CO_2_ and show an abnormal [Ca^2+^]_cyt_ pattern in guard cells [22]. This altered pattern of [Ca^2+^]_cyt_ in CO_2_/HCO_3_^−^-treated guard cells prompted us to design a screening method to identify genes implicated in [Ca^2+^]_cyt_ regulation during stomatal response to CO_2_.

AEQ photoprotein has been extensively used in the Ca^2+^ signaling field for almost 40 years. Because it is convenient, fast, sensitive, easy to use, and applicable to real-time measurement of [Ca^2+^]_cyt_ changes, we chose an AEQ-based system for our genetic screen. According to our data showing that the CO_2_/HCO_3_^−^-induced increase of [Ca^2+^]_cyt_ happened no more than 1 s after CO_2_/HCO_3_^−^ application, almost climbed to the highest value, then dropped almost back to the baseline; the whole process only lasted for about 3 s (Fig. 1a, right), suggesting that the variation of [Ca^2+^]_cyt_ happened both early and rapidly in this physiological process. To identify components underlying this response, *Arabidopsis* mutants were usually isolated by analyzing their leaf temperature through thermal imaging. This traditional method was convenient and common, however thermal imaging takes hours to reach a steady state before detection, which may miss some important components that function earlier in the response to CO_2_.

According to a previous report about detecting stomatal responses to bicarbonate, 1 mM KHCO_3_ has been used for screening [2]. As shown in Figs. 1, 2, both significantly increased bioluminescence and remarkable stomatal closure can be detected at this concentration. Before the screen, a period of dark treatment for AEQ incubation is necessary, so we conducted another preliminary test because dark can influence guard cell status. We found that even in dark treatment, 1 mM KHCO_3_ still can cause closure of the stomata (Fig. 2b), further suggesting the suitability of this screening method.

By using this AEQ-based method and treatment with 1 mM KHCO_3_, we obtained *mci* and *mcs* mutants from about 35,000 M_2_ seeds. We will continue to analyze these mutants and characterize the function of these genes. This series of experiments will shed light on the mechanism of calcium-mediated CO_2_/HCO_3_^−^ response in the guard cell, which appears to occur early during CO_2_/HCO_3_^−^-induced stomatal closure.

## Conclusions

We have developed a sensitive method for isolating stomatal CO_2_/HCO_3_^−^ response genes that function early in the response and play a role in regulating [Ca^2+^]_cyt_ transient changes. This method will be helpful in elucidating the Ca^2+^-dependent regulation of stomatal response.

## Methods

### Plant material and growth conditions

Lines of *Arabidopsis thaliana* ecotype *Col*-*0* constitutively expressing the intracellular Ca^2+^ indicator AEQ (pMAQ2; a gift from Marc R. Knight) or Cameleon (YC3.6; a gift from Simon Gilroy) were used. Plants homozygous for the AEQ-transgenic *Arabidopsis* plant were selected from the second generation after transformation (T1 plants). One such plant, expressing a high level of AEQ, was selected for subsequent experiments.

Plants were grown in soil or in medium containing Murashige and Skoog salts (MS; PhytoTechnology Laboratories), 3% (w/v) sucrose (Sigma), and 0.6% agar (Solarbio) in controlled environmental rooms at 20 ± 2 °C. The fluency rate of white light was ~ 80–100 μmol m^−2^ s^−1^. The photoperiod was 16 h light/8 h dark. Seeds were sown on MS medium, placed at 4 °C for 3 days in the dark, and then transferred to growth rooms.

### AEQ bioluminescence-based Ca^2+^ imaging

[Ca^2+^]_cys_ was measured using *Arabidopsis* plants expressing AEQ. Leaves (1 per well) were treated evenly with 150 μL of 10 μM coelenterazine (Sigma, C2230) in 96-well white culture plates 4 to 6 h before imaging and placed in the dark and in the glass chamber to remove CO_2_. AEQ bioluminescence imaging was performed using a Berthold LB985 system equipped with a light-tight box and a cryogenically cooled, back-illuminated CCD camera. The recording of luminescence was started 30 s prior to treatment and lasted for 5 min. All the treatments were carried out in the dark, and the experiments were carried out at room temperature (22–24 °C).

Similarly, guard cells were used for AEQ bioluminescence imaging. Rosette leaf epidermal peels from 3- to 4-week-old plants were placed in a microwell chamber in incubation buffer for 4–6 h in the dark. AEQ bioluminescence imaging of guard cells was performed using a bioluminescence microscope (Sclis; Biocover) equipped with a light-tight box and a cryogenically cooled, back-illuminated CCD camera. The recording of luminescence was started 60 s prior to treatment and lasted for 5 min. Bright-field images were taken after AEQ imaging. All treatments were carried out in the dark, and the experiments were carried out at room temperature (22–24 °C).

### Mutant screening

*Arabidopsis* seeds expressing AEQ were mutagenized with EMS as described previously [23]. Briefly, about 5000–10,000 seeds were imbibed overnight and then shaken in 0.3% EMS (v/v) for 15 h. The M_1_ seeds were rinsed thoroughly with tap water, sterilized with 10% bleach for 30 min, and washed with sterilized water 5–8 times. M_2_ seeds were harvested separately from individual M_1_ plants. For screening, M_2_ seeds were individually planted in soil and grown for 3 weeks. Leaves from M_2_ plants were placed in a 96-well plate and 100 µL of freshly prepared incubation buffer was added to each well. Kinetic luminescence measurements were performed with an automated microplate luminescence reader (LB960; Berthold) every 0.2 s. After 3 s of luminescence counts, 100 µL of 2 mM KHCO_3_ solution was automatically injected into each well to obtain a final concentration of 1 mM. Bioluminescence was recorded for 30 s per well.

### Stomatal aperture bioassay

Leaves of 3- to 4-week-old seedlings were used in the stomatal aperture assays [24]. Leaves were detached before the light period started. For monitoring stomatal response to KHCO_3_ in light or dark, whole leaves were incubated in stomatal buffer and then exposed to light (100 μmol m^−2^ s^−1^) or dark for 2 h at 25 °C in the glass chamber.

The stomatal buffer contained 50 mM KCl, 0.1 mM CaCl_2_, and 10 mM 2-(N-morpholino) ethanesulfonic acid (MES), adjusted to pH 7.0 with Tris (hydroxymethyl) aminomethane (Tris) [25, 26]. Two hours later, the effects of 1 mM KHCO_3_ on stomatal closure were tested. For characterization of stomatal response mutants, 3 mM KHCO_3_ was also used. Prior to measuring the stomatal aperture, the adaxial epidermis and mesophyll layers were gently separated, and the epidermal strips were placed on microslides containing a drop of stomatal buffer with the desired concentration of KHCO_3_ and covered with coverslips. Pictures of stomata were acquired using an inverted microscope (IX73; Olympus) at 40× magnification. Approximately 100 stomatal apertures from different leaves of each plant type were measured using Image J software (Broken Symmetry Software), and three independent experiments were performed.

### Cameleon-based [Ca^2+^]_cys_ imaging in guard cells

The wild-type plants constitutively expressing GFP fluorescence resonance energy transfer (FRET)-based Ca^2+^ sensor YC3.6, and 10 homozygous lines were generated. Rosette leaf epidermal peels from 3-week-old plants were placed in a microwell chamber in the stomatal buffer for 2 h under light (100 μmol m^−2^ s^−1^). Epidermal peels were treated with 1 mM KHCO_3_ and ratiometric Ca^2+^ imaging was performed using a confocal microscope (LSM710; Zeiss) as described previously [27]. The YC3.6 Ca^2+^ sensor was excited with the 458 nm line of the argon laser. The cyan fluorescent protein (CFP; 473–505 nm) and FRET-dependent Venus (562–536 nm) emission were collected using a 458 nm primary dichroic mirror and the Meta detector of the microscope. Emission images (562–536 nm and 473–505 nm) of epidermal peels were taken, and ratiometric images before and 10 s after addition of 1 mM KHCO_3_.

### Identification of *MCI1* and *MCS1* by MutMap analysis

We backcrossed *mci1* or *mcs1* to AEQ-expressing *Col*-*0* and produced F_2_ individuals. Plants with the *mci1* or *mcs1* phenotype were then subjected to MutMap analysis to find the mutated gene [28]. DNA of 30 F_2_ progeny showing the mutant phenotype was isolated and then bulked using an equal amount of DNA from each plant. This bulked DNA was then subjected to MutMap analysis.

## Data Availability

The raw data from all experiments as well as the material used in this manuscript can be obtained from the corresponding authors upon reasonable request.

## Abbreviations

CAs: Carbonic anhydrases
SLAC1: SLOW ANION CHANNEL-ASSOCIATED 1
PATROL1: A MUNC 13 ortholog in *Arabidopsis* controls the tethering of an H^+^-ATPase
ALMT12: ALUMINUM-ACTIVATED MALATE TRANSPORTER 12
RHC1: RESISTANT TO HIGH CO_2_
OST1: OPEN STOMATA 1
HT1: HIGH LEAF TEMPERATURE 1
ABA: Abscisic acid
[Ca^2+^]_cyt_: Free calcium ion concentration in cytosol
AEQ: Aequorin photoprotein
*Mci*: Mutant of HCO_3_^−^/CO_2_insensitive
*Mcs*: Mutant of HCO_3_^−^/CO_2_sensitive
EMS: Ethyl methane sulfonate
CAs: Carbonic anhydrases
GCA2: GROWTH CONTROLLED BY ABSCISIC ACID 2
